# Ultrasonic Signal Processing Method for Dynamic Burning Rate Measurement Based on Improved Wavelet Thresholding and Extreme Value Feature Fitting

**DOI:** 10.3390/mi16030290

**Published:** 2025-02-28

**Authors:** Wenlong Wei, Xiaolong Yan, Juan Cui, Ruizhi Wang, Yongqiu Zheng, Chenyang Xue

**Affiliations:** Key Laboratory of Instrumentation Science and Dynamic Measurement Ministry of Education, North University of China, Taiyuan 030051, Chinacuijuan@nuc.edu.cn (J.C.);

**Keywords:** adaptive thresholding, adjustable wavelet threshold function, low SNR, precise echolocation, ultrasonic measurement

## Abstract

Ultrasonic measurement techniques are increasingly used to measure the burning rates of solid rocket fuel, but challenges arise due to noise and signal attenuation caused by the motor’s multi-layered structure. This paper proposes an adaptive thresholding method combined with a wavelet threshold function for effective ultrasonic signal denoising. Additionally, an extreme value feature fitting algorithm is introduced for accurate echo signal localization, even in low signal-to-noise ratio (SNR) conditions. Numerical simulations show a 10 dB improvement in SNR at −20 dB, with a correlation coefficient of 0.83 between the denoised and true signals. Echo localization tests across 12 SNR levels demonstrate a consistent error below 1 μs. Compared to other algorithms, the proposed method achieves higher precision, with a maximum displacement error of 0.74 mm. Hardware-in-the-loop experiments show an increase in SNR from −15 dB to 5.78 dB, with maximum displacement and rate errors of 0.9239 mm and 0.781 mm/s. In fuel-burning experiments, the burning rate curve closely matches the theoretical curve, with an initial fuel thickness error of only 0.12 mm, confirming the method’s effectiveness in complex environments.

## 1. Introduction

Solid motors, as the propulsion systems for rockets, are chosen primarily for missiles and rocket propulsion due to their simple structure and ease of use. The burning rate of the solid motor is a key parameter that reflects the combustion of the internal propellant and provides the thrust, which can directly affect rocket performance [1,2,3]. Therefore, accurate measurement of the burning rate of solid motors is of significant importance for their design and analysis. The measurement of rocket motor burning rates currently relies primarily on dynamic testing methods, which involve periodic and continuous measurements of the motor to obtain more precise data. Common dynamic testing methods include the Crawford Bomb Method, high-speed photography, and ultrasonic measurement. The Crawford Bomb Method is limited by environmental factors and the precision of target-line installation, which makes it challenging to achieve accurate results. High-speed photography requires the motor casing to be modified with windows, potentially compromising the motor’s structural integrity, and it is sensitive to lighting conditions and background interference, which can affect the accuracy of the results [4]. In contrast, ultrasonic measurement is widely used for dynamic burning rate tests due to its high sensitivity, high spatial resolution, nondestructive nature, and low cost [5,6,7]. Additionally, ultrasonic testing technology is also employed in biomedical imaging, defect detection, and materials evaluation. This paper explores the application of ultrasonic technology for the dynamic measurement of motor burning rates. (The detailed explanation is presented in Section S1 of the Supplementary Materials).

Ultrasonic sensors apply the principle of TOF to measure distance, which computes the travel time of ultrasonic echo reflected from the target [8,9]. However, the calculation of TOF is affected by two factors. On the one hand, low SNR results in unclear echo signal characteristics, making it difficult to perform subsequent echolocation calculations. On the other hand, for the denoised signal, there is no suitable method to accurately locate its true starting point. Scholars have explored various approaches to address these issues. In terms of echo signal denoising, Figueiredo et al. used a cross-correlation algorithm based on ultrasonic reflection echoes to analyze the cross-correlation of two sets of ultrasonic echo signals and obtain the flight time of ultrasound [10]. However, under low SNR conditions, this method may lead to errors due to poor signal similarity. Liu et al. proposed an adaptive FIR filter based on a multi-layer linear neural network to improve the SNR of ultrasonic echo signals [11], but this approach faces convergence issues. Li et al. introduced a novel method using ultrasonic array signal processing to calculate the flight time of ultrasound [12]; although this method achieves high accuracy in low-noise conditions, it is cumbersome, requires significant computational resources, and still struggles to produce accurate results in high-noise situations.

In terms of echo signal localization, Jiang et al. proposed a signal processing method based on a mathematical model of echo signals [13]. By comparing the collected echo data with the established mathematical model of ultrasonic echo, the starting point of the echo signals was located and then the ultrasound flight time was determined. However, this method is less suitable for signals heavily affected by noise, as noise can cause severe distortion of collected echo signals, making it difficult to accurately determine the starting point of the echo signals. Wang et al. proposed an algorithm based on variable threshold and zero-crossing detection; this method set the thresholds that varied with maximum amplitude changes of echo signals. By distinguishing the peak points of the echo signals through the variable thresholds, the localization of the echo signals was realized [14]. However, this method has limitations in dealing with rapidly changing peak values of echo signals, which restrict its application in the burning rate measurement of rocket motors with rapid changes in propellant thickness. Mu et al. proposed an echo signal localization method based on the waveform characteristics of echoes. This method identifies the intersection of the linear fitting of peaks that are closest to the gradient peak points of the upper and lower envelopes of the echo signal as the echo localization point. However, under conditions of low SNR, it is difficult to find fitting feature points that meet the required criteria [15]. Lu et al. used short-time Fourier transform (STFT) to estimate the flight time of ultrasound [16]. Although STFT is an effective time-frequency analysis method, its resolution in time and frequency is closely related to the selected window function and its width, limiting its flexibility in handling signals with different time-frequency characteristics.

In summary, current ultrasonic signal processing methods struggle to simultaneously achieve signal extraction and echo localization under ultra-low SNR conditions [17]. To address this challenge, this study proposes a signal processing method that integrates denoising and echo localization used for the effective extraction and precise localization of echo signals in high-noise environments. For echo signal denoising, an improved wavelet thresholding method is proposed. By improving wavelet threshold functions and threshold selection rules, the algorithm is better suited to the current testing environment, achieving optimal denoising performance; and for echo signal localization, this paper proposes an extreme value feature fitting algorithm based on the waveform characteristics of ultrasonic echo signals. The algorithm accurately determines the starting position of the echo by identifying feature points in the signal and fitting a feature line. By combining these two methods, precise echo localization under ultra-low SNR conditions can be achieved. The numerical simulation results indicate that the algorithm is capable of signal denoising and echo positioning at an extremely low SNR (−22 dB). Experiments conducted with 12 different SNRs, each repeated 10 times, show that the maximum time error of the algorithm is less than 1 μs. Moreover, the hardware-in-the-loop simulation tested the dynamic changes of the water level, with the maximum deviation from the standard water level displacement curve being 0.9239 mm and the maximum deviation from the level changing rate curve being 0.781 mm/s. Finally, actual measurements were conducted on the motor ignition process. The experimental results demonstrate that this algorithm can achieve precise calculation of the burning rate curve, which aligns well with the theoretical burning rate curve. Above all, the experimental results indicate that this algorithm can effectively extract ultrasonic echo signals from intense noise environments and accurately determine the starting position of the echoes. Consequently, this algorithm can be well applied in the field of dynamic measurement of rocket motor burning rates.

## 2. Ultrasonic Measurement Model

**Figure 1:**
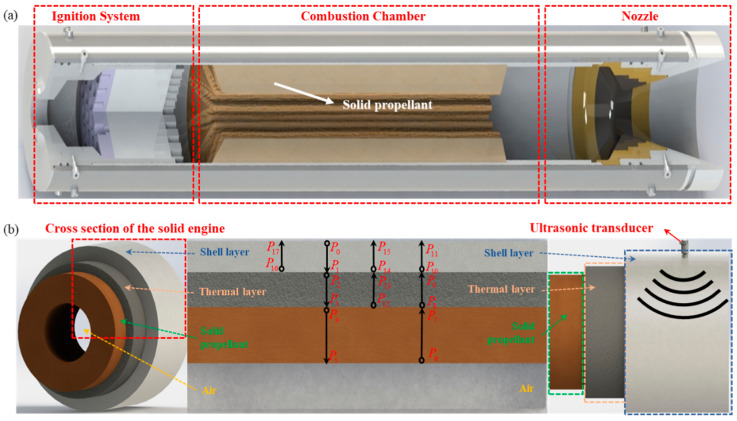
Schematic diagram of rocket motor structure. (**a**) Profile view of a rocket motor. (**b**) Diagram of sound wave propagation path.

During the process of using ultrasound to test the displacement of the rocket motor’s combustion surface, weak echo signals and background noise interference are the main causes of low SNR. The weak echo signals are primarily due to the gradual attenuation of energy as the sound waves pass through multiple layers of media. To analyze the ultrasonic echo attenuation in the multi-layer shells and materials of the solid rocket motor, the ultrasonic measurement model is established for revealing the propagation characteristics of ultrasound in multi-layer medium. The multi-layer structure of the rocket motor is illustrated in Figure 1a. 

During the burning rate test, ultrasound passes successively through the outer shell, insulation, and propellant of the motor, penetrating into the central air cavity. When the ultrasound passes from one medium to another, reflection occurs due to the difference in acoustic impedance between different mediums. The primary cause of energy attenuation and loss during propagation is the energy loss due to multiple reflections. Some of the energy is reflected back during propagation, while the remaining energy continues to transmit forward. As the waves pass through multiple layers of different media, the number of reflections increases, leading to a reduction in the sound wave energy when it finally reaches the medium being tested. Additionally, this portion of the sound wave travels back along the reverse path to the ultrasonic sensor, where it undergoes further energy loss during the return journey, resulting in a weak echo signal received by the sensor.

Therefore, the attenuation of the sound waves primarily results from reflection loss during propagation, while the echoes originate from transmission through each layer. To analyze the effects of transmission and reflection, the transmission and reflection of ultrasound in the multi-layer medium can be described by the transmission coefficient and reflection coefficient, which characterize how wave energy is distributed into transmitted and reflected waves when encountering interfaces between two different medium [18]. The reflection coefficient represents the proportion of sound wave energy that is reflected at the interface and can be calculated based on the difference in acoustic impedance. For the case of normal incidence of sound wave on the motor shell, the reflection coefficient R can be calculated as follows:

(1) $$R_{1}=\frac{Z_{2}-Z_{1}}{Z_{2}+Z_{1}},\;R_{2}=\frac{Z_{3}-Z_{2}}{Z_{3}+Z_{2}},\;R_{3}=\frac{Z_{4}-Z_{3}}{Z_{4}+Z_{3}}$$

where *R*_1_ represents the reflection coefficient between the shell and the insulation layer, *R*_2_ represents the reflection coefficient between the propellant layer and the insulation layer, and *R*_3_ represents the reflection coefficient between the propellant layer and the air cavity. *Z*_1_, *Z*_2_, *Z*_3_, *Z*_4_ respectively denote the acoustic impedance of the shell, insulation layer, propellant layer, and air, and can be described as the product of the sound speed and density of the material [19]:

(2) $$\left\{\begin{array}{l} Z_{1}=\rho_{1}c_{1}\\ Z_{2}=\rho_{2}c_{2}\\ Z_{3}=\rho_{3}c_{3}\\ Z_{4}=\rho_{4}c_{4} \end{array}\right.$$

Here $c_{1},c_{2},c_{3},c_{4}$ represent the speed of sound in each layer, and $\rho_{1},\rho_{2},\rho_{3},\rho_{4}$ denote the density of each layer. The transmission coefficient can be described as the ratio of the impedance of the transmitted wave to the sum of the impedances of the incident and transmitted waves as follows:

(3) $$T_{1}=\frac{2Z_{2}}{Z_{1}+Z_{2}},\;T_{2}=\frac{2Z_{3}}{Z_{2}+Z_{3}}$$

where *T*_1_ represents the transmission coefficient between the shell and the insulation layer, and *T*_2_ represents the transmission coefficient between the insulation layer and the propellant layer.

According to Equations (1)–(3), it can be observed that when ultrasound impinges vertically on the sample surface, the acoustic pressure at each interface is influenced by the acoustic impedance. If the thicknesses of the respective layers are *d*_1_, *d*_2_, *d*_3_, and the initial acoustic pressure of the emitted ultrasound is *P*_0_, then the acoustic pressure *P_i_* (*i* = 1, …, 17) at different positions along the ultrasonic transmission path can be obtained by Equation (4).

(4) $$\left\{\begin{array}{l} P_{1}=P_{0}e^{-\alpha_{1}d_{1}}\\ P_{2}=P_{1}T_{1}\\ P_{3}=P_{2}e^{-\alpha_{2}d_{2}}\\ P_{4}=P_{3}T_{2}\\ P_{5}=P_{4}e^{-\alpha_{3}d_{3}}\\ P_{6}=P_{5}R_{3}\\ P_{7}=P_{6}e^{-\alpha_{3}d_{3}}\\ P_{8}=P_{7}T_{2}\\ P_{9}=P_{8}e^{-\alpha_{2}d_{2}} \end{array}\right.\;\;\left\{\begin{array}{l} P_{10}=P_{9}T_{1}\\ P_{11}=P_{10}e^{-\alpha_{1}d_{1}}\\ P_{12}=P_{3}R_{2}\\ P_{13}=P_{12}e^{-\alpha_{2}d_{2}}\\ P_{14}=P_{13}T_{1}\\ P_{15}=P_{14}e^{-\alpha_{1}d_{1}}\\ P_{16}=P_{1}R_{1}\\ P_{17}=P_{16}e^{-\alpha_{1}d_{1}} \end{array}\right.$$

In Equation (4), *P_i_* (*i* = 1, …, 17) reflects the acoustic pressure at different positions along the propagation path, where $\alpha_{1},\alpha_{2},\alpha_{3}$ represent the sound attenuation coefficients of each layer material. The acoustic propagation path is shown in Figure 1b, *P*_11_ represents the echo pressure reflected from the burning surface, *P*_15_ represents the echo pressure reflected between the insulation layer and the propellant layer, and *P*_17_ represents the echo pressure reflected between the shell and the insulation layer [20].

Based on the above equations, we can calculate the acoustic pressure at different positions along the propagation path during the testing process. However, to establish a comprehensive acoustic wave propagation model, further analysis of the ultrasonic echo signal is necessary. Ideally, ultrasonic echo signals exhibit Gaussian random characteristics, which means their envelopes follow the Gaussian distribution [21,22,23]. Therefore, the Gaussian decay model of ultrasonic echo can be established based on the following distribution:

(5) $$S(t)=Ae^{-\gamma \pi {\left(t-\tau \right)}^{2}}\sin\left[2\pi f_{c}(t-\tau )+\varphi \right]+N_{w}(t)+N_{c}(t)+N_{p}(t)$$

In Equation (5), *A* represents the amplitude of the signal, γ denotes the bandwidth decay factor, *f_c_* represents the center frequency of the echo signal, τ denotes the time delay, φ represents the initial phase, and *N_w_*(*t*), *N_c_*(*t*), *N_p_*(*t*) represents white noise, colored noise, and pulse noise, respectively, which are used to simulate environmental noise interference, ignition interference, and rocket vibration interference in real-world situations. Since the echo signal amplitude is directly proportional to the acoustic pressure, *A* can be replaced with *P* in the model.

## 3. Ultrasonic Echo Signal Processing Method

**Figure 2:**
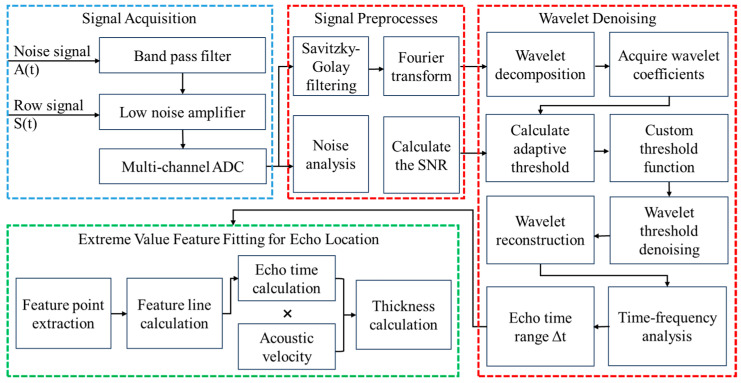
Signal processing flowchart.

The entire signal processing workflow is divided into four main modules: signal acquisition, signal preprocessing, wavelet denoising, and extreme value feature fitting for echolocation. As shown in Figure 2. In the signal acquisition module, the system captures external noise and echo signals through two channels, providing input data for subsequent processing. The signal preprocessing module comprises four parts: noise analysis and pre-filtering of the signal. Noise analysis calculates the SNR of the current frame, providing input parameters for the next step’s adaptive threshold. Pre-filtering includes Savitzky–Golay smoothing filtering and Fourier transform, which are used to remove impulsive pulse noise and colored noise from the raw signal, respectively. The wavelet threshold denoising module receives the preprocessed signals and SNR parameters, and performs the wavelet decomposition and adaptive threshold calculations. After completing wavelet threshold denoising, the signal is reconstructed, and time-frequency analysis is performed to roughly locate the range of the echo. Finally, the extreme value feature fitting module extracts feature points from the signals processed by wavelet denoising and fits them to determine the starting point of the echo. In this way, we are able to achieve highly accurate echo localization, even in environments with low SNRs.

### 3.1. Preprocessing of Ultrasonic Echo Signals

In the actual testing environment, there are three main sources of noise: pulse noise, colored noise, and additive white noise. To sequentially filter out these noises for optimal signal restoration, the signal preprocessing section first eliminates the colored noise. During the rocket motor burning rate test, the ultrasound signal used has a high and fixed resonant frequency, while the echo signal has a short duration. In contrast, the low-frequency vibration signals generated by the rocket motor during the test persist throughout the entire test cycle and have a relatively narrow frequency range. To effectively extract the ultrasonic signal from the test data and remove low-frequency colored noise, the frequency spectrum of the current frame signal is analyzed based on Fourier transform. An adaptive FIR band-stop filter is constructed to filter out interference signals outside the ultrasonic frequency range.

Specifically, let the Fourier transform of the original signal be *S*(*f*), where f represents the frequency. In the frequency domain, a band-stop filter *H*(*f*) is constructed and multiplied by the original spectrum *S*(*f*). The filtered frequency-domain signal *S*′(*f*) is then obtained, and subsequent inverse transformation gives the filtered time-domain signal *S*′(*t*). The mathematical representation of this process is as follows in Equation (6):

(6) $$\left\{\begin{array}{l} S\left(f\right)=\int_{-\infty }^{+\infty }\bar{S}(t)e^{-j2\pi t}dt\\ S^{\prime }(f)=\bar{S}(f)H(f)\\ S^{\prime }(t)=\int_{-\infty }^{+\infty }S^{\prime }(f)e^{2\pi jft}df \end{array}\right.$$

For the processing of impulsive pulse signals, the Savitzky–Golay method is a commonly used smoothing filter. Due to its configurable window length and fitting order, it can be applied to specifically target the signal for processing. In this paper, considering the data volume and boundary point handling, a window length of 5 and a fitting order of 3 are configured [24], as shown in Equation (7) (the detailed explanation is presented in Section S2 of the Supplementary Materials):

(7) $$\left\{\begin{array}{l} \bar{y_{-2}}=\frac{1}{70}\left[69y_{-2}+4y_{-1}-6y_{0}+4y_{1}-y_{2}\right]\\ \bar{y_{-1}}=\frac{1}{35}\left[2y_{-2}+27y_{-1}+12y_{0}-8y_{1}+2y_{2}\right]\\ \bar{y_{0}}=\frac{1}{35}\left[-3y_{-2}+12y_{-1}+17y_{0}+12y_{1}-3y_{2}\right]\\ \bar{y_{1}}=\frac{1}{35}\left[2y_{-2}-8y_{-1}+12y_{0}+27y_{1}+2y_{2}\right]\\ \bar{y_{2}}=\frac{1}{70}\left[-y_{-2}+4y_{-1}-6y_{0}+4y_{1}+69y_{2}\right] \end{array}\right.$$

where *y_i_* (*i* = −2, −1, …, 2) represents the 5 data points before smoothing, and $\bar{y_{i}}(i=-2,-1,\ldots ,2)$ represents the data after Savitzky–Golay smoothing. The Savitzky–Golay smoothing can effectively remove irregular abrupt pulse noise of the echo signals while preserving the original characteristics of the signal. The smoothed signal is defined as.

After filtering out the pulse noise and colored noise, the remaining signal primarily contains additive white noise as interference. In the subsequent wavelet denoising process, the SNR is used as an adaptive threshold parameter. Therefore, the remaining noise signal needs to be analyzed during preprocessing. In the rocket motor testing environment, noise is superimposed in an additive manner, and additive white noise can be directly measured by the noise sensors. Thus, the SNR of the signal to be processed can be approximately calculated as follows in Equation (8):

(8) $$SNR=10lg\left[\sum_{n=0}^{N-1}\bar{s^{2}}(n)-N_{w}^{2}(n)/\sum_{n=0}^{N-1}N_{w}^{2}(n)\right]$$

### 3.2. Wavelet Thresholding Denoising

During the preprocessing, pulse noise and colored noise have already been filtered out. To better restore the true signal, further denoising of the signal $\bar{s}(t)$ is required. In this section, wavelet transform is used for its analysis. For a continuous-time signal $\bar{s}(t)$, its wavelet transform is defined as follows in Equation (9):

(9) $$\omega_{i}=\frac{1}{\sqrt{\left|a\right|}}\int_{-\infty }^{\infty }\bar{s}(t)\bar{\psi (\frac{t-b}{a})}dt$$

where *a* is the scale factor, *b* is the translation factor, and $\bar{\psi (t)}$ is the conjugate of the wavelet basis function. In this paper, the chosen basis function is the Meyer wavelet, and $\omega_{i}=[\omega_{1},\omega_{2},\ldots ,\omega_{n}]$ represents the wavelet coefficients after decomposition [25].

The key to wavelet threshold denoising lies in the selection of threshold points and threshold functions, which correspond to different handling methods of the coefficient. For traditional threshold functions, the hard thresholding function directly removes all the coefficients and noise below a specified threshold, resulting in discontinuities of the signals, while the soft threshold function preserves the smoothness and continuity of the signal better, which may lead to loss of signal details and blurring [26]. However, the issue arises because the threshold function’s curve near the threshold point is either too flat or too steep, leading to either insufficient or excessive processing of the signal around the threshold point. To address these issues, an improved threshold function is proposed for wavelet threshold denoising. This threshold function can adjust the steepness of the curve before and after the threshold point, and it also allows for the adjustment of the vertical coordinate of the threshold point, significantly increasing the flexibility of its use. The improved threshold function is expressed as follows:

(10) $$\omega =\left\{\begin{array}{ll} \mathrm{sgn}(\omega )\times \left(\omega -\frac{\lambda }{1+\sigma }\times \gamma^{\sqrt{\omega^{2}-\lambda^{2}}}\right) & \left|\omega \right|>\lambda \\ \mathrm{sgn}(\omega )\times \frac{\sigma }{1+\sigma }\times e^{\eta \times \left(\left|\omega \right|-\lambda \right)}\times \left|\lambda \right| & \left|\omega \right|\leq \lambda \end{array}\right.$$

In Equation (10), λ represents the threshold point, $\omega^{\prime }$ represents the output wavelet coefficient, ω represents the input wavelet coefficient, η,σ,γ represents the adjustment factor of the threshold function, and η≥10,σ>0,0<γ<1.

**Figure 3:**
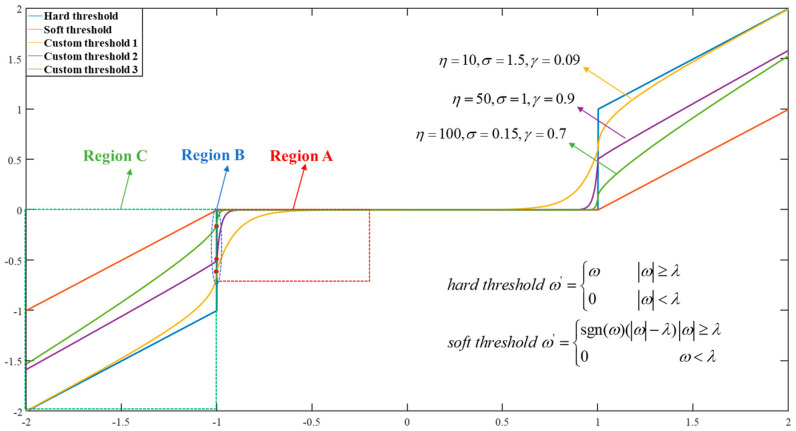
Comparison of wavelet threshold functions.

The threshold function is shown in Figure 3. As a comparison, the traditional hard threshold function and soft threshold function are presented and plotted. In contrast, the custom threshold function provides more flexibility by allowing the processing near the threshold point to be adjusted according to the actual situation. The three regions A, B, and C in the figure are controlled by the parameters *η*, *σ*, and *γ*, respectively. The parameters *η* and *γ* are used to control the steepness of the curves in regions A and C before and after the threshold point. When *η* is smaller, the curve in region A becomes flatter, and when *γ* is smaller, the curve in region C becomes steeper. A steeper threshold function means that within the range close to the threshold *λ*, the function value can quickly increase from 0 (or decrease in the negative direction to a certain value), which helps to more accurately preserve the edges and detailed information in the signal. A flatter threshold function, on the other hand, helps reduce overprocessing during the denoising process, allowing the weak components to be better preserved while making the processed signal more natural and smoother. 

The selection of the parameter σ directly determines the vertical coordinate of the threshold point. A larger *σ* leads to a larger absolute value of the vertical coordinate, making it more suitable for signals with higher noise levels. In contrast, for signals with lower noise levels, *σ* should be set smaller. In this paper, the application scenario involves ultrasonic echo signals, which are weak and have a high frequency. Therefore, a small value of *η* is chosen because important weak components and detailed information exist before the threshold point. Additionally, *γ* is set smaller since more high-frequency echo information needs to be preserved after the threshold point. Given the high noise level in this testing environment, *γ* can be adjusted to a larger value.

In wavelet threshold denoising, the selection of the threshold λ also critically affects the denoising performance. This study focuses on the continuous measurement of objects with varying thicknesses. As the object gradually becomes thinner over time, the amplitude of ultrasonic echoes will gradually increase. Therefore, within the same noise environment, the SNR of the echoes collected during different transmission periods varies. Traditional threshold selection methods, such as fixed thresholds, are not suitable in this scenario.

To improve the denoising effect on signals with changing SNR, the threshold point should be able to adaptively and dynamically adjust. In this study, the unbiased likelihood estimation threshold method is employed and improved. The SNR is used as a parameter to adjust the threshold, aiming to calculate thresholds under different SNR conditions. The calculation process is illustrated by Equations (11)–(13).

Take the absolute value of the wavelet coefficients, and arrange them in ascending order, and then square each element to obtain the new estimator $\varpi_{i}$ as follows in Equation (11):

(11) $$\varpi_{i}=[\varpi_{1},\varpi_{2},\ldots ,\varpi_{n}]$$

In Equation (8), *n* denotes the number of wavelet coefficients in the decomposition. Additionally, a risk vector $R_{i}$ is defined as follows in Equation (12):

(12) $$R_{i}=\frac{\left[n-2i+(n-i)\varpi_{i}+\sum_{k=1}^{i}\varpi_{k}\right]}{n}$$

where for i=1,2,…,n, the minimum value *R_min_* from the risk vector *R_i_* is taken as the risk value, with the corresponding wavelet coefficient $\omega_{\min}$.

Since the SNR of each frame of the collected signal changes, in order to adaptively adjust the threshold for each frame’s wavelet denoising, the SNR is introduced as a parameter for adjusting the threshold. The SNR is already obtained in the preprocessing stage; therefore, the improved unbiased estimation threshold can be expressed in Equation (13):

(13) $$\lambda =\sqrt{\frac{\omega_{\min}}{{\left(1+\sqrt{e^{SNR}}\right)}^{2}}}$$

By combining the wavelet threshold function proposed above with the wavelet threshold point, the wavelet coefficients after decomposition can be processed to obtain the threshold-denoised coefficients $\omega_{i}^{\prime }=[\omega_{1}^{\prime },\omega_{2}^{\prime },\ldots ,\omega_{n}^{\prime }]$. Reconstructing these coefficients provides the denoised time-domain signal $\hat{S}(t)$. Time-frequency analysis of this signal allows for the determination of the time range ΔT of the echo signal. To accurately locate the starting point of the echo signal for precise measurement, further localization of the echo’s starting point will be performed within the time range ΔT based on the signal $\hat{S}(t)$.

### 3.3. Extreme Value Feature Fitting Algorithm

Although the time range of the echo is determined, it is necessary to further pinpoint the specific timing of echo occurrence within the time range ΔT. Therefore, the extreme value feature fitting algorithm is proposed, which can identify the feature points and feature lines of the echo signals to calculate the precise moment of echo occurrence. 

**Figure 4:**
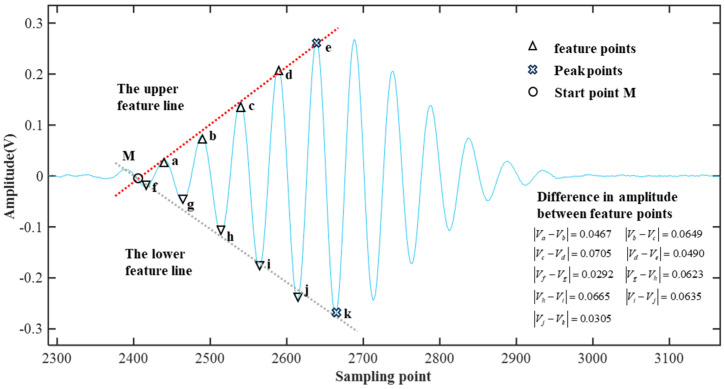
Schematic diagram of the characteristic line method.

As shown in Figure 4, through feature analysis of the original echo signal under low SNR conditions, we observe that both the upper and lower envelopes of the echo signal contain a maximum peak and a minimum peak, respectively. Additionally, there are several extremum points preceding these peaks, with the amplitudes of adjacent extremum points approximating an arithmetic progression. Therefore, the maximum peak and the extremum points preceding it can be selected as feature points, and similarly, the minimum peak and the extremum points preceding it can also be selected as feature points. Subsequently, linear functions are used to fit these feature points on both sides, resulting in two characteristic lines. The intersection of these two characteristic lines can be used to determine the starting point of the echo.

As shown in Figure 4, *e* and *k* represent the peak points of the upper and lower envelopes, respectively. We select the peak point of the upper envelope and the four preceding extremum points *a*, *b*, *c*, *d*, and *e* as feature points. Similarly, we select the peak point of the lower envelope and the five preceding extremum points *f*, *g*, *h*, *i*, *j*, and *k* as feature points. Linear functions are then fitted to these feature points on both sides, and the intersection point *M* of the fitted lines on the upper and lower sides is used as the starting point of the echo signal. 

The coordinates of the intersection point *M* of the upper and lower feature lines can be obtained as follows in Equation (14):

(14) $$x=\frac{b_{2}-b_{1}}{k_{1}-k_{2}}\;y=k_{1}\frac{b_{2}-b_{1}}{k_{1}-k_{2}}+b_{1}$$

As the start point of echo is the intersection of the upper and lower feature lines, the coordinates of the start point can be located as (*x*, *y*). Accordingly, the corresponding horizontal coordinate *x* is considered as the start position of the echo. By multiplying this point by the sampling time interval, the start time of the echo can be calculated.

## 4. Numerical Simulation Experiment

To evaluate the reliability and superiority of the algorithm, numerical simulations are conducted using MATLAB v.R2016a on the mathematical model *S*(*t*) in Equation (5). The discrete sampling interval of the samples is T = 0.01 μs, resulting in a total of 10,000 sampling points over a duration of 100 μs. To simulate real-world environmental interference, pulse noise *N_p_*(*t*), colored noise *N_c_*(*t*), and white noise *N_w_*(*t*) are introduced into the signal. The parameters of each layer in the simulation model are set as specified in Table 1.

**Figure 5:**
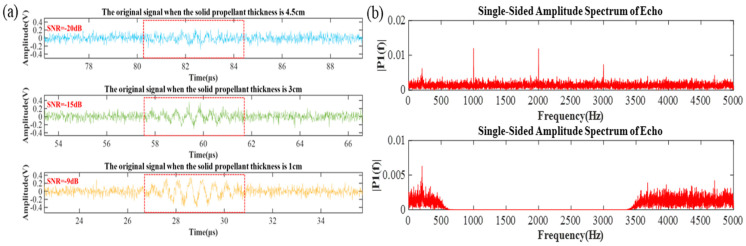
(**a**) Echo signals at different propellant thicknesses. (**b**) Spectrum diagram of echo signal when the propellant thickness is 4.5 cm.

According to the mathematical model, Table 1 provides the parameters for each layer of material. To simulate the variations of the rocket motor propellant in the actual measurement environment, three different propellant thicknesses at different time points were set for the simulation. Based on the relationship between the speed of sound and propellant thickness in the table, the true values of the echo starting times at different propellant thicknesses can be calculated as 80.45 μs, 57.37 μs, and 26.6 μs, respectively (the detailed explanation is presented in Section S1 of the Supplementary Materials). By substituting the parameters from the table into Equations (1)–(5), pulse noise, white noise, and colored noise with a frequency band of 0.5~3.5 kHz were introduced. Numerical calculations were performed on the simulation results, as shown in Figure 5. As shown in Figure 5a, the simulation results of the original echo signals $S_{1}(t),S_{2}(t),S_{3}(t)$ for three different propellant thicknesses are presented. The figure shows, from top to bottom, the propellant thicknesses of 4.5 cm, 3 cm, and 1 cm, respectively.

Under the same testing environment, the background noise at different time points remains largely consistent. However, as the propellant thickness decreases, the attenuation of the sound waves gradually weakens, and the echo amplitude increases. Therefore, the SNR of the echo signals gradually improves as the propellant thickness decreases. The SNRs of the three echoes in the figure are −20 dB, −15 dB, and −9 dB, respectively.

To validate the effectiveness of the algorithm, the echo signal $S_{1}(t)$ with the lowest SNR of −20 dB is selected for illustration, and its spectrum is analyzed. Figure 5b, the first image, shows the frequency spectrum of this signal. This plot displays the frequency band containing colored noise, into which colored noise at frequencies of 1 kHz, 2 kHz, and 3 kHz has been introduced. To achieve noise removal in the preprocessing stage, a 100-order FIR filter designed with a Hamming window is first used to filter out the colored noise. The second image shows the result of noise removal in this frequency band. It is evident from the figure that, after FIR filtering, the introduced colored noise is effectively removed, and the filtered signal provides a solid foundation for the subsequent processes. 

**Figure 6:**
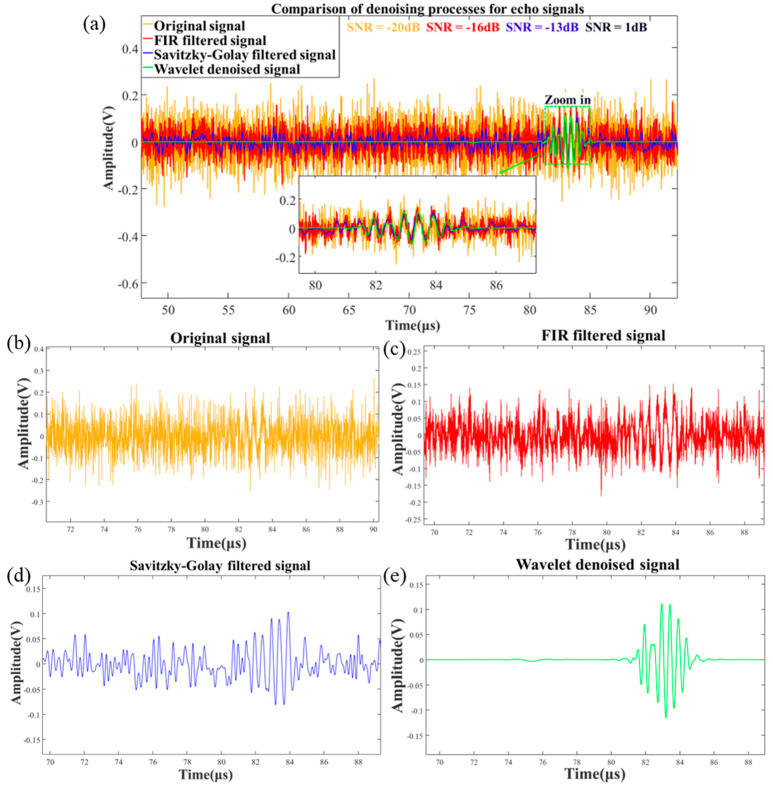
(**a**) Comparison of denoising processes for echo signals. (**b**) Original signal. (**c**) FIR filtered signal. (**d**) Savitzky–Golay smoothed signal. (**e**) Wavelet denoised signal.

According to the algorithm proposed in this paper, the original signal is first preprocessed and then subjected to wavelet denoising. In the preprocessing stage, the first step is to filter out colored noise and pulse noise, followed by wavelet denoising to remove white noise. Figure 6 illustrates the signal-filtering process in the simulation.

Figure 6a shows a comparison of the denoising results at each step of the numerical simulation process. The golden curve represents the original signal $S_{1}(t)$; the red curve represents the signal $S^{\prime }(t)$ after FIR filtering, with an SNR of −16 dB; the blue curve represents the signal $\bar{S}(t)$ after applying Savitzky–Golay smoothing filtering to $S^{\prime }(t)$, with an SNR of −13 dB; and finally, the green curve represents the wavelet threshold-denoised signal $\hat{S}(t)$, with an SNR of 10 dB. From the figure, it is clear that the signal quality improves gradually throughout the denoising process, with the SNR increasing by 30 dB from start to finish. This comparison demonstrates that the proposed algorithm provides effective denoising for the signal. 

Furthermore, to evaluate the signal reconstruction accuracy, the correlation coefficient is used as a metric to compare the three different denoised signals with the noise-free pure signal set in the simulation. The definition of the correlation coefficient is as follows:

(15) $$NCC=\frac{\sum_{i=1}^{N}s_{i}{s^{\prime }}_{i}}{\sqrt{\sum_{i=1}^{N}s_{i}^{2}\sum_{i=1}^{N}{s^{\prime }}_{i}^{2}}}\;\left(-1<NCC<1\right)$$

**Figure 7:**
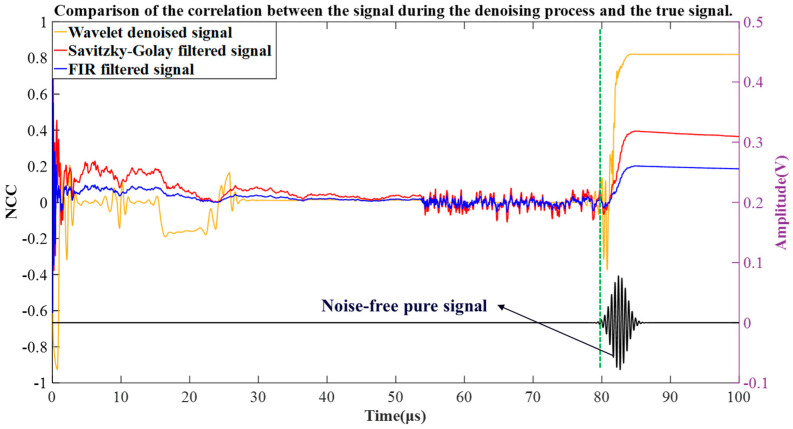
Signal correlation comparison diagram.

As shown in Figure 7, the waveform at the bottom represents the noise-free echo signal $S_{p}(t)$, while the three curves above it represent the variation of the correlation coefficient between the signals $S^{\prime }(t),\bar{S}(t),\hat{S}(t)$, and $S_{p}(t)$ over time. It can be observed that the correlation coefficient curve exhibits large fluctuations at the initial time due to the initial pulse interference. The Savitzky–Golay smoothing filter struggles to handle the pulse noise at the start, resulting in significant fluctuations in the correlation between $S^{\prime }(t),\bar{S}(t),\hat{S}(t)$ and $S_{p}(t)$. Between 55~80 μs, due to the inability of the FIR filter and Savitzky–Golay smoothing filter to effectively filter signals around short-duration non-stationary components, the correlation coefficient curve shows small rapid jitter. However, the correlation coefficient of signal $\hat{S}(t)$ maintains a steady trend during this period.

Subsequently, the correlation coefficient curve shows a sharp change at 80 μs. This is because the signal components before 80 μs only contain background noise, which has a low correlation with the pure signal. However, during the time period from 80 μs to 85 μs, it can be observed that an echo signal appears in the signal components, causing a sharp increase in the correlation. Furthermore, the maximum correlation coefficients between the signals $S^{\prime }(t),\bar{S}(t),\hat{S}(t)$ and $S_{p}(t)$ are 0.83, 0.4, and 0.2, respectively. From the correlation comparison results, it can be concluded that the SNR of the signal gradually improves during the denoising process, and the final filtered signal exhibits a higher similarity to the real signal. 

Following the aforementioned steps, the final denoised signals $\hat{s_{1}}(t),\hat{s_{2}}(t),\hat{s_{3}}(t)$ are obtained. In order to locate the positions of the echoes, it is necessary to perform time-frequency analysis on these signals. By examining the frequency characteristics of the echo signals, the corresponding time ranges of the echo signals can be identified.

**Figure 8:**
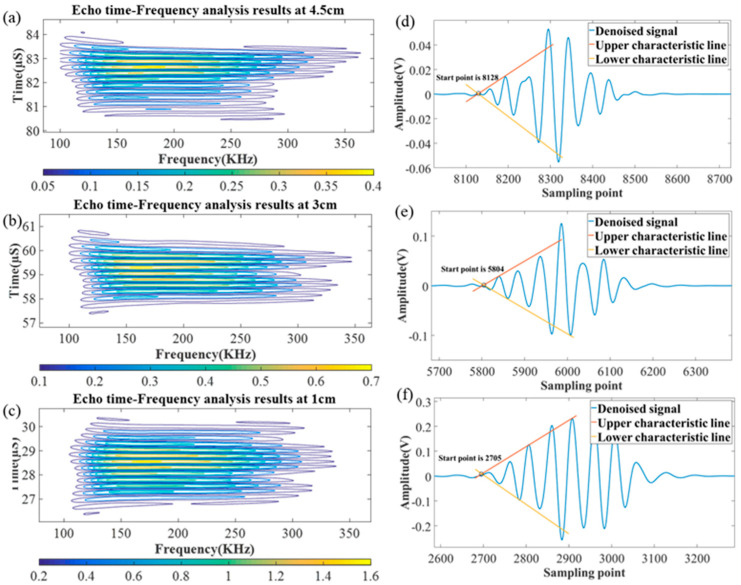
Time-frequency analysis and echo localization results. (**a**) Echo time-frequency analysis results at 4.5 cm. (**b**) Echo time-frequency analysis results at 3 cm. (**c**) Echo time-frequency analysis results at 1 cm. (**d**) Echo localization results at 4.5 cm. (**e**) Echo localization results at 3 cm. (**f**) Echo localization results at 1 cm.

Figure 8a–c shows the time-frequency analysis results of the denoised echo signals for the three different propellant thicknesses. The color-distributed areas in the spectrograms represent the results of the time-frequency analysis. Since the resonant frequency of the sound wave used in the simulation is 200 kHz, the times corresponding to this frequency band in the figure represent the time range of the echo signal and are ΔT_1_ = 26.3~30.5, ΔT_2_ = 57.3~61, ΔT_3_ = 80.3~85. To determine the exact moments of the echoes, the proposed extreme value feature fitting algorithm is performed by calculating the feature lines of each echo signal. The locating echoes within the denoised signal are depicted in Figure 8d–f; the three graphs correspond to the time-frequency analysis results on the left side. As shown in Figure 8d, within the time range, the extremum features are fitted, and the intersection of the characteristic lines indicates the starting point of the echo signal. The position of this point is 8128, and with a sampling interval of 0.01 μs, the starting time of this echo signal is 81.28 μs. Using the same analysis, the echo starting times for the other two graphs, Figure 8e,f, are determined to be 58.04 μs and 27.05 μs, respectively. Comparing the numerical simulation results with the preset true echo times (80.45 μs, 57.37 μs, and 26.6 μs), the errors are 0.83 μs, 0.67 μs, and 0.45 μs, respectively. It can be observed that the error increases with the distance of the echo position. This is because a greater distance corresponds to a thicker measured object, resulting in a smaller echo amplitude. Consequently, in the same noise environment, the signal quality is poorer, leading to increased errors in echo positioning.

Through the above simulation, the echo starting times for three different signals were calculated. By multiplying the echo starting times by the speed of sound, the corresponding propellant thicknesses for each signal were determined to be 44.46 mm, 29.56 mm, and 9.70 mm (with errors of 0.54 mm, 0.44 mm, and 0.30 mm, respectively). This process is extended to the entire test cycle, assuming an initial thickness of 4.5 cm, a test cycle duration of 55 seconds, and a single test interval of 1 second. Under the condition of uniform propellant thickness change over the entire test period, the preset simulation true values are used. Then, following the above steps, noise is added to the 55 frames of echo data, and the results processed by different echo localization algorithms yield the subsequent propellant recession curve.

**Figure 9:**
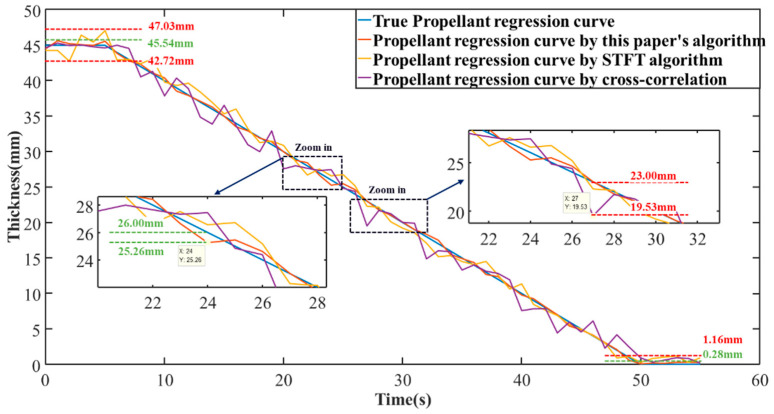
Simulation results of propellant surface regression.

As shown in Figure 9, the propellant recession curves calculated using different echo localization algorithms are presented. The image is divided into three sections: the 0~5 s time period simulates the initial stationary state, the 50~55 s time period simulates the final stationary state, and the 5~50 s period represents the ignition test process, during which the propellant thickness decreases uniformly. By comparing the echo localization algorithm proposed in this paper with two other commonly used echo localization algorithms, it can be observed that the algorithm proposed in this paper achieves the highest accuracy in echo localization, followed by the STFT, while the cross-correlation algorithm performs the worst. In the figure, the red dashed line represents the error range of the other algorithms, while the green dashed line represents the error range of the proposed algorithm. During the initial stationary stage, the maximum error of the proposed algorithm is only 0.54 mm, while the maximum error of the other two algorithms reaches 2.28 mm. In the final stationary stage, the proposed algorithm almost coincides with the preset true value, with a maximum error of only 0.28 mm, while the other two algorithms show a maximum error of 1.16 mm. In comparison, during the combustion phase, the propellant recession curves obtained using the cross-correlation and short-time Fourier transform algorithms show significant fluctuations with large variations, with a maximum error of 3.47 mm, whereas the propellant recession curve obtained using the proposed algorithm has much smaller fluctuations, with a maximum error of 0.74 mm.

**Figure 10:**
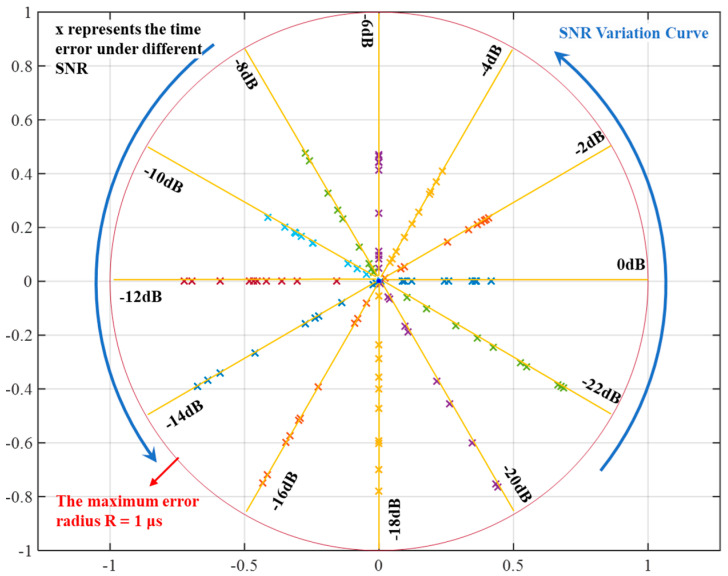
The time error of the algorithm at different SNRs.

To further validate the effectiveness of the algorithm in handling echo signals under different SNRs, experiments are conducted at the same thickness with varying SNRs. The experimental error results are presented in Figure 10.

The experiment was configured with 12 SNRs, ranging from 0 dB to −22 dB, decreasing in steps of 2 dB, and 10 trials were conducted for each SNR. The distance from each intersection point to the center represents the time error. It can be observed that the results of the 10 experiments under the same SNR are quite similar, demonstrating good consistency of the algorithm. Additionally, as the SNR decreases, the experimental error gradually increases, but it does not exceed the outer circle with an error radius of 1 μs. This indicates that the proposed algorithm has superiority in processing signals under extremely low SNRs.

## 5. Experiment and Discussion

**Figure 11:**
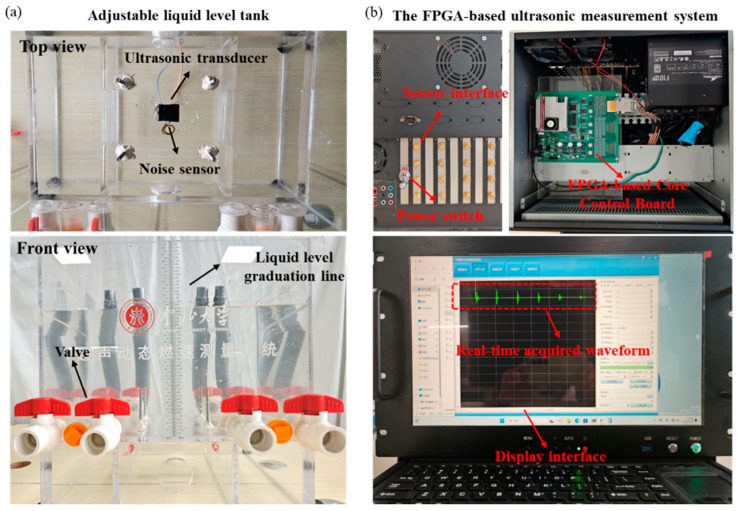
Experimental testing system. (**a**) Adjustable liquid level tank. (**b**) The FPGA-based ultrasonic measurement system.

To verify the practical effectiveness of the proposed algorithm, the FPGA-based dynamic ultrasonic testing system was established, using water as a substitute for solid propellants to simulate the dynamic changes of the burning surface in a motor. The experimental testing system is shown in Figure 11.

The specific workflow of the system is as follows: During operation, the ultrasonic transducer is installed at the bottom of the water tank, where the water level decreases as the electric valve is adjusted. The hardware system generates a 1 MHz excitation pulse each 50 ms to drive the ultrasonic transducer and generate ultrasound. The ultrasonic waves propagate through the water, and then reflect back as ultrasonic echo signals when they reach the interface with air. Simultaneously, in the FPGA, the delay control module completes counting and notifies the ADC sampling control module to sample the echo signals at a rate of 20 Msps. Finally, the sampled data are transmitted to the hard drive of the PC via the PCIE drive control module of FPGA for storage, signal processing, and displaying the result.

### 5.1. Hardware-in-the-Loop Simulation Experiment

**Figure 12:**
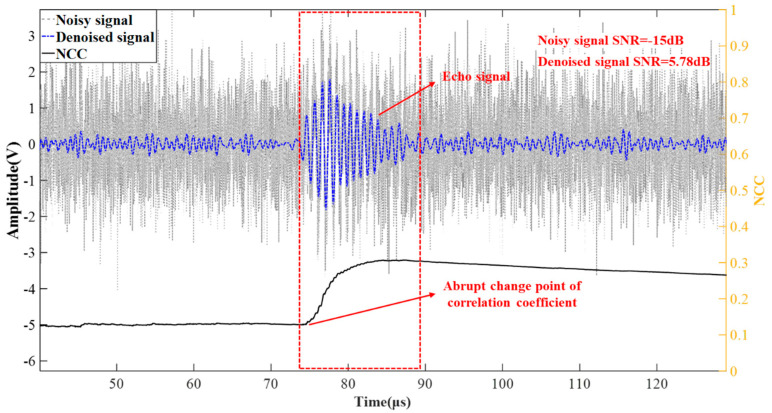
Comparison of denoising effects on measured signals.

The final experimental results should consist of two parts: signal denoising results and water surface descend results. These are derived from the calculation of echo positions. The experiment is conducted in a high-noise environment, where ultrasound is transmitted every 50 ms. Any frame of sampled data is selected as the echo signal to be processed. At this point, the SNR of the noisy signal is −15 dB. The denoised signal is shown in Figure 12.

Figure 12 contains three curves: the noisy signal, the denoised signal, and the correlation coefficient curve. It is evident that the signal quality significantly improves after denoising, with the SNR increasing from −15 dB to 5.78 dB. The black curve below represents the variation of the correlation coefficient between the two signals over time. At the position where the echo appears, the correlation coefficient is suddenly changed and continues to increase. Until the echo disappears, the correlation coefficient curve gradually declines. Therefore, by analyzing the correlation coefficient curve, the correlation between the original signal and the denoised signal can be obtained, and the approximate starting time of the echo can also be estimated based on the abrupt changes in the curve.

**Figure 13:**
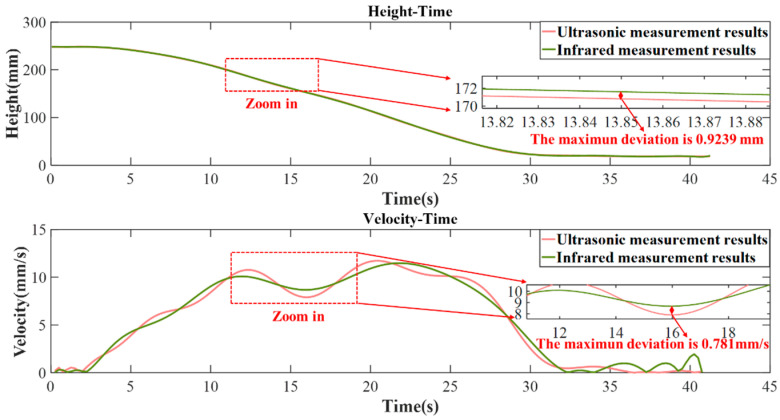
Results of dynamic water surface descent testing.

After determining the echo propagation time, multiplying it by the sound speed of the liquid (1848 m/s) yields the current liquid level height. The system conducts periodic measurements every 50 ms, and as the liquid level drops, the position of the echo reception moves forward. By combining the sound speed with each measurement, the liquid level height is calculated, allowing for the plotting of a height-time curve and further analysis to derive the rate of change curve, as shown in Figure 13. To assess the testing accuracy of this method, an infrared thickness gauge is used during the experiment for synchronous testing. The testing cycle is 10 ms and the entire test duration is 42 s.

The experimental results are illustrated in Figure 13. The initial water level was 245 mm. From 0 to 2.5 s, the valve remained closed, and the water level stayed relatively constant. However, due to external vibrations, the liquid surface exhibited slight fluctuations, which were also reflected in minor oscillations in the changing rate curve during this period. Between 2.5 and 5 s, the valve gradually opened, at this point the internal water pressure was at its highest, causing a rapid decrease in the liquid surface, with the changing rate quickly rising from 0 to 5 mm/s.

From 5 to 12 s, the valve was fully open, leading to a progressive increase in the water level’s rate of descent. Between 12 and 16 s, the valve was gradually closed, resulting in a decreasing trend in the changing rate. After 16 s, multiple valves were simultaneously opened, and the changing rate increased again, continuing until 22 s, when the liquid level dropped to 100 mm and the water pressure decreased. This decrease in water pressure led to a subsequent decline in the changing rate curve. By 32 s, the liquid level nearly ceased to drop and stabilized at a height of 20 mm. Since the valve diameter was larger than the liquid level at this point, the fluid passing through the valve caused vortex shedding due to the Kármán vortex street phenomenon (cavitation), resulting in liquid surface vibrations. Correspondingly, the changing rate curve also displayed fluctuations during this time. The test concluded at 42 s. 

Comparing the measured results with those from the infrared thickness gauge shows that the water level displacement curves of the two kinds of results are nearly identical, with only minor differences in some areas; these differences stem from the different measurement cycles and different calculation methods of the two. The infrared thickness gauge has a shorter measurement interval, allowing it to capture changes over a shorter period of time. The rate of change curves, derived from the derivative of the displacement curves, amplifies the differences in velocity within a unit period. Overall, both of the two curves exhibit similar trends, with significant differences observed between 32 and 42 s. These differences are attributed to the smoothing of long-duration oscillation data performed by the infrared thickness gauge. From 2.5 to 32 s, the maximum deviation in the changing rate curves between the two is 0.781 mm/s, while the maximum deviation in the displacement curves is 0.9239 mm.

### 5.2. Real-World Experimental Results

To further validate the applicability and effectiveness of the proposed algorithm, an on-site test of the rocket motor ignition process was conducted using the measurement system described above. The propellant thickness of the rocket motor was 191.6 mm, with a total ignition duration of 10.72 s. During the ignition process, the system conducted tests with a firing cycle of 5 ms.

**Figure 14:**
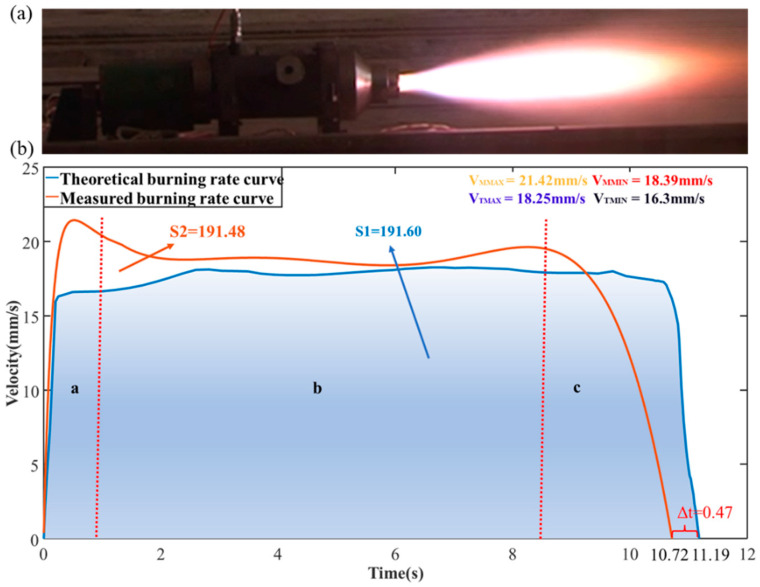
(**a**) Motor ignition test site. (**b**) Measured burn rate curve of rocket motor.

The test results are shown in Figure 14b. The blue curve represents the estimated theoretical burn rate curve, while the red curve represents the measured burn rate curve. The image is divided into three regions: a, b, and c. Region a represents the initial ignition phase, where the measured burn rate rapidly increases to 21.42 mm/s within the first second due to rapid pressurization inside the chamber. Subsequently, due to gradual pressure equilibrium, the burn rate stabilizes in region b. However, there are slight fluctuations in the burn rate curve due to minor changes in internal temperature, with the minimum measured burn rate during this phase being 18.39 mm/s. The system then enters the third phase, as shown in region c, where the fuel is nearly exhausted and the chamber volume increases, which in turn boosts the burn rate, which then rapidly drops to 0 mm/s. Comparing the measured burn rate curve with the theoretical burn rate curve shows a similar trend, with significant differences only in regions a and c. By analyzing the on-site test data, it was found that these differences were due to changes in the fuel density inside the chamber, leading to a lower theoretical burn rate calculation. Given the known initial propellant thickness of 191.6 mm, the theoretical burn time was estimated to be 11.19 s, which is 0.47 s longer than the measured burn time. Additionally, the maximum value of the theoretical burn rate curve is 18.25 mm/s, and the non-zero minimum value is 16.3 mm/s. The maximum errors between the measured and theoretical values are ΔV_MAX_ = 3.17 mm/s and ΔV_MIN_ = 2.09 mm/s. To further validate the accuracy of the measured results, the integral of the burn rate curve was calculated to determine the initial propellant thickness. The calculated initial propellant thickness was 191.48 mm, with a deviation of only 0.12 mm from the true value. From the comparison results, it can be concluded that the proposed algorithm provides high testing accuracy.

## 6. Conclusions

This paper proposed an improved adaptive wavelet thresholding algorithm and echo localization algorithm to address challenges for the precise extraction and localization of weak echo signals at low SNR and high noise interference. Numerical simulations show that the algorithm improves the SNR from −20 dB to 10 dB, with a correlation coefficient of 0.83. The echo time localization error is under 1 μs across various SNR levels. Compared to similar algorithms, the proposed method achieves the smallest fuel surface displacement error, with a maximum error of 0.74 mm. In hardware loop simulations and motor tests, the proposed algorithm demonstrates robust performance, with rate change curves closely matching the reference values. The maximum errors are 0.781 mm/s and 2.09 mm/s, validating the algorithm’s precision and reliability in complex environments. Further improvements will be focused on weak signal recovery by optimizing the wavelet basis function. For example, in Figure 6e, defects in the initial segment of the denoised signal are attributed to the weak signal and the wavelet basis function not fully matching the echo signal characteristics. Nevertheless, the method proposed in this paper demonstrates promising results in processing time-varying and non-stationary signals with low SNRs, similar to ultrasonic echoes. This denoising approach can also be applied to fields such as seismic signaling and radar signal processing.

## Figures and Tables

**Table 1:** Simulation parameter.

Layer	Density	α	v	d_1_	d_2_	d_3_
Shell	7.82 g/cm^3^	0.055 db/cm/MHz	5800 m/s	0.025 m	0.025 m	0.025 m
Insulation	1.20 g/cm^3^	0.15 db/cm/MHz	1540 m/s	0.002 m	0.002 m	0.002 m
Propellant	1.02 g/cm^3^	0.18 db/cm/MHz	1300 m/s	0.045 m	0.03 m	0.01 m

α represents the attenuation coefficient, v represents the speed of sound, and d_1_, d_2_, and d_3_ represent the thicknesses of the respective layers at different time points.

## Data Availability

All the necessary data are included in the article.

## Supplementary Materials

The following supporting information can be downloaded at: https://www.mdpi.com/article/10.3390/mi16030290/s1, Section S1. The principle of ultrasonic measurement. Section S2. Savitzky-Golay smoothing filter. Figure S1. Schematic Diagram of Ultrasonic Measurement Waveform; Table S1. Simulation parameter.
