# Supplementary material for: Ultrasonic Signal Processing Method for Dynamic Burning Rate Measurement Based on Improved Wavelet Thresholding and Extreme Value Feature Fitting
Source: Micromachines (Basel). 2025 Feb 28;16(3):290. doi: 10.3390/mi16030290 (PMC11945480; doi:10.3390/mi16030290)
Supplement: Supplementary file 1 [file micromachines-16-00290-s001.zip › Supporting Information.pdf]

Supporting Information

Signal processing method for rocket motor ultrasonic burning rate measurement based on improved wavelet thresholding and extreme value feature fitting

Wenlong Wei, Xiaolong Yan*, Juan Cui, Ruizhi Wang, Yongqiu Zheng, Chenyang Xue

a. The key Laboratory of Instrumentation Science & Dynamic Measurement Ministry of Education, North University of China, Taiyuan, 030051, China.

* Corresponding author.

E-mail address: yanxl@nuc.edu.cn

Section S1. The principle of ultrasonic measurement

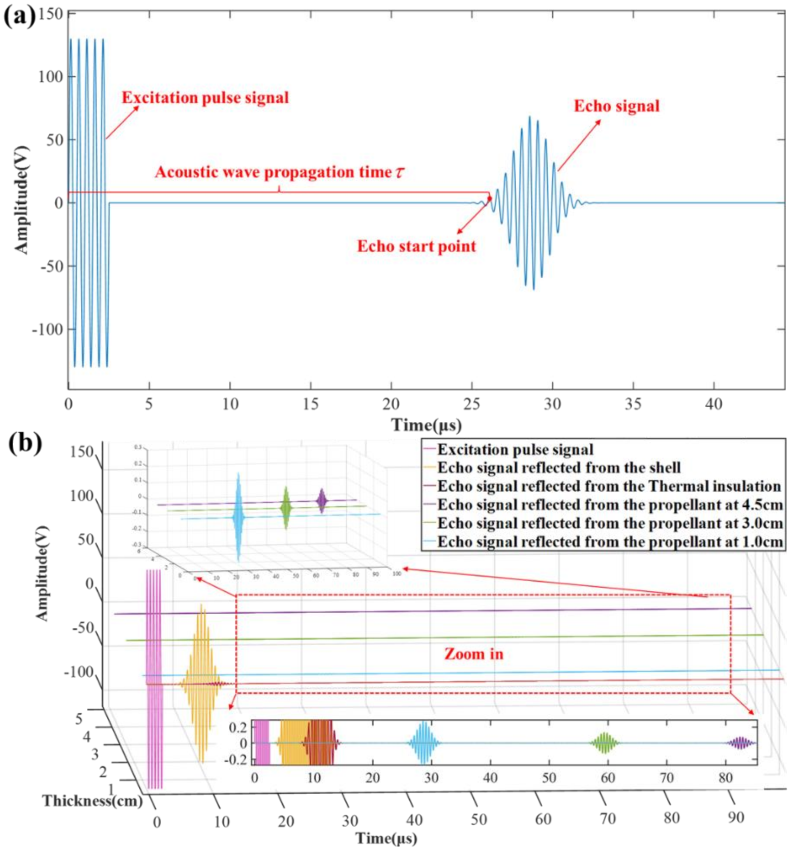

Figure S1. Schematic Diagram of Ultrasonic Measurement Waveform

The principle of ultrasonic thickness measurement is to calculate the time of flight (TOF) of the ultrasonic wave. By combining this with the known propagation speed of the ultrasonic wave in the medium, we can determine the current thickness of the measured medium. As shown in Fig. S1(a), the figure illustrates the excitation waveform and the echo signal, where the TOF is τ. Once the acoustic wave propagation time is calculated and multiplied by the sound speed, the current thickness

of the measured object can be obtained, as shown in the following calculation formula:

$$D = \frac{c \times \tau}{2} \tag{1}$$

Where c represents the sound speed, and D indicates the thickness of the measured object. The division by 2 in the formula accounts for the fact that the ultrasonic wave must travel to the object and back.

This paper aims to measure the rate of change of the propellant burn surface in a rocket motor. By periodically measuring the variation in propellant thickness and then differentiating with respect to time, we can determine the combustion rate of the propellant. The burning rate r can be calculated using the following formula:

$$r = \frac{\Delta L}{\Delta t} \tag{2}$$

Where Δt is the measurement period, and Δl is the difference in thickness between two consecutive measurements. When the measurement period Δt is small, r can be regarded as the instantaneous burn rate. The simulation parameters set in this paper are shown in Table S1.

Table S1. Simulation parameter

	Material Density	Attenuation Coefficient	Acoustic Velocity	Initial Thickness	Mid-thickness	Ending Thickness
Shell Layer	$7.82 g/cm^3$	0.055db/cm/MHz	5800m/s	0.025m	0.025m	0.025m
Insulation Layer	$1.20 g/cm^3$	0.15db/cm/MHz	1540m/s	0.002m	0.002m	0.002m
Propellant Layer	$1.018 g/cm^3$	0.18db/cm/MHz	1300m/s	0.045m	0.030m	0.010m

As shown in Table S1, the parameters of each layer of materials set in this paper allow for the calculation of the TOF of the acoustic wave using Equation (1) as follows:

$$\begin{aligned} T_1 &= \frac{2D_1}{c_1} \\ T_2 &= \frac{2D_1}{c_1} + \frac{2D_2}{c_2} \\ T_3 &= \frac{2D_1}{c_1} + \frac{2D_2}{c_2} + \frac{2D_3}{c_4} \end{aligned} \tag{3}$$

Where T_1 represents the time of flight of the echo from the outer shell, T_2 represents the time of flight of the echo from the insulation layer, and T_3 represents the time of flight of the echo from the propellant. Based on the parameters in the table, we can calculate T_1=8.6μs and T_2=11.2μs. Since the echo time only varies with the thickness of the measured material, the TOF for the echoes from the outer shell and the insulation layer remains constant. This characteristic is shown in the waveform diagram in Fig. S1(b), where the unchanged position of the waveforms allows for easy differentiation during the signal processing.

On the other hand, the thickness of the propellant gradually decreases with combustion time. The paper uses three different thicknesses of propellant for illustration, and based on the data in the table, the corresponding time of flight calculated using the above parameters is 80.45 μs, 57.37 μs, and 26.6 μs, respectively. The corresponding echoes are shown in Fig. S1(b).

Section S2. Savitzky-Golay smoothing filter

Savitzky-Golay filtering is a mathematical processing technique used for smoothing data. It preserves high-frequency features of the data while reducing noise by fitting a polynomial to subsets of data points. This filtering method, based on local polynomial least squares fitting, achieves the purpose of smoothing and fitting data by performing local polynomial fitting within a sliding window and replacing the original signal with the fitted polynomial. The computational steps for this algorithm are as follows:

(1) Select a window containing an odd number of data points.

(2) Within this window, fit a polynomial of degree k using the least squares method.

(3) Calculate the value of this polynomial at the center point of the window, and this value serves as the filtered result.

This process is repeated across the entire dataset, resulting in a smoothed curve. In practical applications, choosing the appropriate window size and polynomial degree is crucial, as too large a window or too high a polynomial degree can lead to over-smoothing of the data, while too small a window or too low a polynomial degree may not effectively remove noise. In this paper, a window size of 5 data points and a polynomial degree of 3 were selected for fitting the data points within the window.

Divide the collected data into 2n+1 nodes ranging from x_{-n} , x_{-m} ,…, x_{-1} , x_0 , x_1 ,…, x_m , x_n , with the corresponding data values for each node are y_{-n} , y_{-m} ,…, y_{-1} , y_0 , y_1 ,…, y_m , y_n . Set h as the equal interval sampling step size, and perform the transformation. This can transform the aforementioned 2n+1 equally spaced points into: $t_{-n}=-n, t_{-n+1}=-n+1, \dots, t_{-1}=-1, t_0=0, t_1=1, \dots, t_{n-1}=n-1, t_n=n$.

Fit the obtained experimental data with an m degree polynomial. Let the fitting polynomial be:

$$y(t)=a_0+a_1x+\dots+a_mx^m \tag{4}$$

Wherein, the undetermined coefficients in Equation (4) are determined using the least squares method. Let:

$$\sum_{i=-n}^{n} R_i^2=\sum_{i=-n}^{n}\left[\sum_{j=0}^{m} a_j t^j{}_i - y_i\right]^2=\phi(a_0,a_1,\cdots a_m) \tag{5}$$

To minimize $\phi(a_0,a_1,\cdots a_m)$, we take the partial derivatives of these expressions with

respect to $a_j(j=0,1,\ldots,m)$, and set them equal to zero. This set a normal Equations is:

$$\sum_{n=-N}^{N} y_n t_n^k = \sum_{j=0}^{m} a_j \sum_{n=-N}^{N} t_n^{i+j} \tag{6}$$

When n=2 (i.e., 5 nodes) and m=3, a system of normal equations will be obtained. By solving this system, we can solve a_0, a_1, a_i, a_{it} and substitute them into Equation (6). Then, by setting t=0, 1, −1, 2, −2, we can derive the five-point cubic smoothing formula as follows:

$$\begin{cases} \overline{y_{-2}} = \frac{1}{70}\left[69y_{-2} + 4y_{-1} - 6y_0 + 4y_1 - y_2\right] \\ \overline{y_{-1}} = \frac{1}{35}\left[2y_{-2} + 27y_{-1} + 12y_0 - 8y_1 + 2y_2\right] \\ \overline{y_0} = \frac{1}{35}\left[-3y_{-2} + 12y_{-1} + 17y_0 + 12y_1 - 3y_2\right] \\ \overline{y_1} = \frac{1}{35}\left[2y_{-2} - 8y_{-1} + 12y_0 + 27y_1 + 2y_2\right] \\ \overline{y_2} = \frac{1}{70}\left[-y_{-2} + 4y_{-1} - 6y_0 + 4y_1 + 69y_2\right] \end{cases} \tag{7}$$

Where y_i (i=-2， -1,…,2) represents the 5 points data before smoothing, and $\overline{y_i}$ represents the data after Savitzky-Golay smoothing.
